# Soft Self‐Healing Robot Driven by New Micro Two‐Way Shape Memory Alloy Spring

**DOI:** 10.1002/advs.202305163

**Published:** 2023-11-20

**Authors:** Xianrong Liang, Chenggang Yuan, Chaoying Wan, Xiaolong Gao, Chris Bowen, Min Pan

**Affiliations:** ^1^ Department of Mechanical Engineering University of Bath Bath UK; ^2^ International Institute for Nanocomposites Manufacturing (IINM) WMG University of Warwick Coventry CV4 7AL UK; ^3^ College of Mechanical and Electrical Engineering Beijing University of Chemical Technology Beijing 100029 China

**Keywords:** micro two‐way shape memory alloy spring, self‐healing elastomers, self‐healing robots, soft robots

## Abstract

Soft robotic bodies are susceptible to mechanical fatigue, punctures, electrical breakdown, and aging, which can result in the degradation of performance or unexpected failure. To overcome these challenges, a soft self‐healing robot is created using a thermoplastic methyl thioglycolate–modified styrene–butadiene–styrene (MG‐SBS) elastomer tube fabricated by melt‐extrusion, to allow the robot to self‐heal autonomously at room temperature. After repeated damage and being separated into several parts, the robot is able to heal its stiffness and elongation to break to enable almost complete recovery of robot performance after being allowed to heal at room temperature for 24 h. The self‐healing capability of the robot is examined across the material scale to robot scale by detailed investigations of the healing process, healing efficiency, mechanical characterization of the robot, and assessment of dynamic performance before and after healing. The self‐healing robot is driven by a new micro two‐way shape‐memory alloy (TWSMA) spring actuator which achieved a crawling speed of 21.6 cm/min, equivalent to 1.57 body length per minute. An analytical model of the robot is created to understand the robot dynamics and to act as an efficient tool for self‐healing robot design and optimization. This work therefore provides a new methodology to create efficient, robust, and damage‐tolerant soft robots.

## Graphical abstract

Soft robotic bodies are susceptible to mechanical fatigue, punctures, electrical breakdown, and aging. A soft self‐healing robot is created using a thermoplastic MG‐SBS elastomer tube fabricated by melt‐extrusion, to allow the robot to self‐heal autonomously at room temperature. After repeated damage and being separated into several parts, the robot is able to heal and enable almost complete recovery of robot performance.

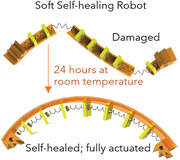

## 1 Introduction

Soft robots are composed of compliant materials that are capable of flexible movement, autonomous behavior, and safer human‐machine interaction. Inspired by biological systems, such systems are designed to mimic the locomotion and performance of soft animals that exist in nature to achieve complex motion in uncertain or constrained environments. In recent decades, a variety of soft robotic systems and devices have been developed,^[^ ^1^, ^2^, ^3^, ^4^, ^5^ ^]^ with a range of milestone works.^[^ ^6^, ^7^, ^8^, ^9^, ^10^, ^11^, ^12^ ^]^ However, the soft bodies of the robots can be damaged during the operation as a result of mechanical fatigue, punctures, electrical breakdown, and electrochemical corrosion, which can lead to unexpected failure during operation as an associated additional energy consumption and cost. The creation of soft self‐healing robots that are able to self‐repair and heal from structural damage will highly enhance the robustness and sustainability of soft robots.

A range of self‐healing mechanisms and materials are available to embed within a soft robot. An extrinsic self‐healing process relies on the intervention of pre‐embedded healing agents in polymers, often carried in the form of microcapsules or microvascular fibers, which respond to release the healing agents upon rupture and react to bind damaged surfaces. Intrinsic self‐healing of polymer is possible by exploiting the reversible interactions among the dynamic covalent bonds that are produced by associative or dissociative mechanisms, or non‐covalent bonds with or without external stimulus.^[^ ^13^, ^14^, ^15^, ^16^, ^17^, ^18^, ^19^, ^20^, ^21^, ^22^, ^23^ ^]^ Comprehensive reviews on the topic of self‐healing polymers and their applications for soft robotics are published.^[^ ^13^, ^14^, ^15^, ^16^, ^17^, ^18^, ^19^, ^20^, ^21^, ^22^, ^23^ ^]^ Since the intrinsic healing mechanism operates at the molecular level, it has the particular advantage of being able to enable repeated self‐healing of the material.

Non‐covalent interactions are ubiquitous in nature and biological systems, such as hydrogen bonding, metal‐ligand coordination, or ionic interactions, contributing to protein assembly, structural stability, molecular recognition, mechanical properties, and responsive functions. Inspired by nature, the introduction of non‐covalent interactions into commercial polymers provides a unique potential to develop new functionalities and expand the applications of engineering polymers. As an example, commercial poly (styrene‐butadiene‐styrene) elastomers (SBS) typically exhibit phase‐separated microstructures, where the styrene blocks cluster and act as physical crosslinks for reinforcement and melt‐processibility. By exploiting the vinyl groups of the butadiene blocks of SBS, we have successfully grafted a range of polar groups onto the SBS backbone via one‐step thiol‐ene chemistry and introduced greater levels of polarity to the non‐polar elastomer to intrinsically affect both dielectric and mechanical properties, and in particular to introduce a self‐healing function.^[^ ^24^, ^25^, ^26^ ^]^ We have synthesized the methyl thioglycolate grafted SBS (MGSBS) elastomer in our previous work and investigated the reaction conditions including the excess of methyl thioglycolate required for efficient thiol group and vinyl group coupling and minimal interchain crosslinking.^[^ ^24^ ^]^ The excellent self‐healing ability of MGSBS at ambient conditions was demonstrated in the event of mechanical cuts and electrical breakdown.^[^ ^25^ ^]^ After experiencing a dielectric breakdown, the dielectric strength can be recovered by up to 67% of the initial strength, and after mechanical damage, 39% of the initial dielectric strength can be recovered. Firstly, we punctured the elastomer with a needle probe, and the pinhole defects were cleaned and self‐healed at room temperature. To maximize the degree of damage and healing, the pristine elastomer was cut fully through its thickness using a sharp and clean scalpel and healed by applying a small load of 5 N at ambient temperature for 5 min to ensure the damaged surfaces were in good contact and flattened in‐plane. Once the cutting site had healed, the ≈25 mm in length cutting had fully closed, and the healed site could be clearly observed when stretched normally to the cutting direction. The MGSBS had recovered 25% of its strength with a strain of over 100%. According to our investigations, the highest graft ratio of 98.5% of MGSBS led to the highest self‐healing efficiency and highest relative permittivity.^[^ ^26^ ^]^

Although the range of self‐healing mechanisms of polymers have shown significant potential to empower self‐healing capability to soft robots, only a limited number of publications demonstrate their use in soft robotic devices and applications.^[^ ^17^, ^19^, ^20^ ^]^

A self‐sealing soft gripper that is resistant to punctures was first developed by Shepherd et al. in 2013,^[^ ^27^ ^]^ by embedding highly fibrillated polyaramid fibers in a silicone elastomer. This allows the fabricated soft actuator or gripper to recover its original shape by physically pressing the punctured surfaces and sealing the hole. Terryn et al. developed a variety of soft self‐healable actuators, grippers, and robotic hands based on thermal reversible Diels‐Alder (DA) reactions (11, 28, 29). These devices could be healed and were able to fully recover performance from macroscopic damages such as scratches, cuts, and ruptures after applying a mild heat treatment (70–90 °C). For example, healing of the actuator was demonstrated when damaged under two dramatic phenomena,^[^ ^11^ ^]^ namely (i) perforation damage when the device was over‐pressurized at 0.46 bar and (ii) a deep wall cut of ≈4.4 mm in length and a thickness of 0.3 mm. Both forms of damage were successfully healed after 30 h at a maximum healing temperature of 70 °C. To eliminate the need for external heating resources, a soft self‐healable gripper was developed that used a novel Diels‐Alder network with high molecular mobility, which enabled autonomous healing macroscopic damage at room temperature^[^ ^28^ ^]^; however, this is at the expense of reduced output power and force capabilities of the gripper.

With the recent development of additive manufacturing technology, 3D‐printed self‐healable robotic devices have been developed.^[^ ^29^, ^30^, ^31^ ^]^ Roels et al. tested the healability of a 3D‐printed Diels‐Alder‐based gripper.^[^ ^29^ ^]^ When the fingers of the gripper were subjected to a puncture, cut, and being sliced in half, they could heal after being heat treated at 90 °C for 30 min, and after held at room temperature for 24 h the gripper was able to recover its full performance with only visible scars. Wallin et al. developed a cost‐efficient stereolithography (SLA)‐based fabrication technique to create soft robots.^[^ ^30^ ^]^ They prototyped fluidic elastomer actuators from a silicone (polydimethylsiloxane) material that was filled with thiol‐ene resins and formed at high resolution (≈50 µm) and at rapid fabrication speeds. The material system allowed rapid self‐healing via sunlight‐induced photopolymerization to recover actuator properties after damage from scratches and punctures. When the membrane of the fluidic elastomer actuators was pierced, the resin was able to flow outward and be exposed to sunlight, thereby leading to photopolymerization and self‐healing of the hole. A micro‐stereolithography system was also used to fabricate soft self‐healable muscles, which were manufactured from a disulfide‐based supramolecular network with healing capacities.^[^ ^31^ ^]^ The muscles were able to recover entirely from being cut in half to fully restore their initial structural integrity and mechanical strengths to 100% after healing for 2 h at 60 °C. The muscle was able to lift a weight ten times its own weight for multiple cycles.

To enable shape memory effects, Zhang et al. developed thermo‐reversible polyurethanes (PDAPU) with targeted light‐controlled shape memory and self‐healing properties (32). The reactive cross‐linking of aniline trimer (AT) in the PDAPU network formed a homogeneous network with enhanced mechanical properties, good solvent resistance, and highly efficient photothermal capability. The materials were used to print a variety of self‐healable 3D objects including a butterfly, octopus, hand, and flat dog bone, where the self‐healing efficiency was >70%. Zhang et al. developed a double‐network self‐healing shape memory polymer material for high‐resolution (up to 30 µm) light processing‐based 3D printing technology.^[^ ^33^ ^]^ The material was made by incorporating a semicrystalline polycaprolactone (PCL) into a methacrylate‐based shape memory polymer. The PCL linear polymer provided the self‐healing capability by melting PCL in the matrix at 80 °C, with a healing efficiency of >90% for mechanical damage. A damaged 3D‐printed gripper was able to heal at 80 °C for 5 min. The healed gripper was able to perform when driven by the shape memory mechanism and was able to lift a weight of 10 g. To develop self‐healing modular actuators for reconfigurable robots, Gomez et al. developed a vat photopolymerizable self‐healing elastomer which was capable of extreme elongations of up to 1000%.^[^ ^34^ ^]^ The self‐healing elastomer was developed using a combination of thiol/acrylate mixed chain/step‐growth polymerizations and applied a combination of physical processes and dynamic‐bond exchange via thioethers to achieve full self‐healing capability over multiple damage and healing cycles. Soft modular actuators with complex internal cavities and channel networks can be printed with high resolution and the use of 3D‐printed technology provides an effective fabrication tool to open new research areas in the development of multifunctional soft robots with complex geometries, self‐healing, and shape memory capabilities.

Self‐healable dielectric elastomer actuators (DEAs) have been developed and applied to soft robots to achieve large actuation strain, high bandwidth, high energy density, self‐healing capability, and flexible nature. Zhang et al. reported on the electrical and mechanical self‐healing in high‐performance dielectric actuator which uses a thermoplastic methyl thioglycolate–modified styrene–butadiene–styrene (MG‐SBS) elastomer.^[^ ^25^ ^]^ They investigated the DEAs and characterized the electrical properties and actuator response and healing process before and after healing. The MG‐SBS elastomer shows promising healing capability, which could heal damage from dielectric breakdown, mechanical damage, and a combination of mechanical and electrical damage. This work has benchmarked the feasibility of using MG‐SBS‐based material for creating complex soft robots that provide a range of motion. Soft hydraulically amplified self‐healing electrostatic (HASEL) actuators, which employ a mechanism that couples electrostatic and hydraulic forces to achieve a variety of actuation motions were first developed by Acome et al.^[^ ^35^ ^]^ The HASEL actuators apply a mechanism that couples electrostatic and hydraulic forces to achieve a variety of actuation modes. The electrohydraulic mechanism is used to activate soft‐matter hydraulic architectures, while the fluidic actuation mechanism is used to realize the muscle‐like performance and self‐sensing abilities of DE actuators. The HASEL actuator exhibited muscle‐like performance and self‐healing capability after being subjected to dielectric breakdown. The use of liquid dielectrics in HASEL actuators allows for immediate recovery of functionality after dielectric breakdown events. The actuators can be used in a stacked configuration to generate high deformation and their response can be adapted to different geometries. The group prototyped two HASEL actuators in both donut and planar geometries. A stack of five donut HASEL actuators was able to achieve a 37% linear strain and large actuation response at frequencies up to 20 Hz. A soft gripper was developed using two stack donut HASEL actuators which were able to successfully grasp delicate objects, such as raspberries and eggs. The advantage of using liquid dielectrics is that they can rapidly return to an insulating state after dielectric damage, which enables HASEL actuators to effectively self‐heal after being subjected to dielectric breakdown. The donut HASEL actuators were able to provide self‐healing for 50 dielectric breakdown events, with the highest breakdown voltage of 29.3 kV. To further improve HASEL performance, Tian et al. developed new HASEL actuators that integrate a bilayer polymer shell for improved properties of high dielectric strength, dielectric permittivity, and elastic modulus.^[^ ^36^ ^]^ The new bilayer HASEL exhibited a strain of 164% at 5 kV and a load‐bearing capability of 620 mN at 6 kV. The high strain, high output load, and self‐healing capability position HASEL actuators as promising candidates for the development of multifunctional soft robotic systems.

Tang et al. developed a new class of fully soft self‐healable pumps that utilize electrical energy to pump liquid through an electron and ion migration mechanism.^[^ ^37^ ^]^ A self‐healing liquid based on a dibutyl sebacate‐tung oil solution, where the tung oil was dissolved evenly in the dibutyl sebacate, was used as the liquid medium of the pump. Tung oil was selected as it has excellent solidification properties due to its unique composition, namely oxygen‐linked fatty carboxylate residue and reactive conjugated carbon–carbon double bonds. When the soft pumps were damaged, the self‐healing liquid was exposed to air, and a solid film was formed which solidified, which automatically heals any damage. The self‐healing time is dependent on the temperature, and for a small puncture damage a healing time of ≈6 h at 35 °C and ≈24 h at 24  °C is required. The self‐healable pumps are scalable, which provides opportunities for developing a variety of untethered self‐healable soft robotic devices.

In this work, we have created a new form of soft intrinsically self‐healable robot using a melt‐extruded MG‐SBS elastomer tube, which enables the robot to autonomically self‐heal from multiple damage events at room temperature after 24 h. We examined the self‐healing performance and efficiency of the robot, robot mechanical characterization, and dynamic performance before and after healing. The robot is designed and actuated using a new micro two‐way SMA (TWSMA) spring actuation system. This new approach provides a route to the creation of future soft robots that have large output force, fast locomotion speed, and self‐healing capabilities. We also demonstrated a scalable and continuous melt‐extrusion process for functional soft robotics development.

## 2 Results

### 2.1 Self‐Healing Mechanism and Performance of Melt‐Extruded MG‐SBS Tubes

**Figure 1:**
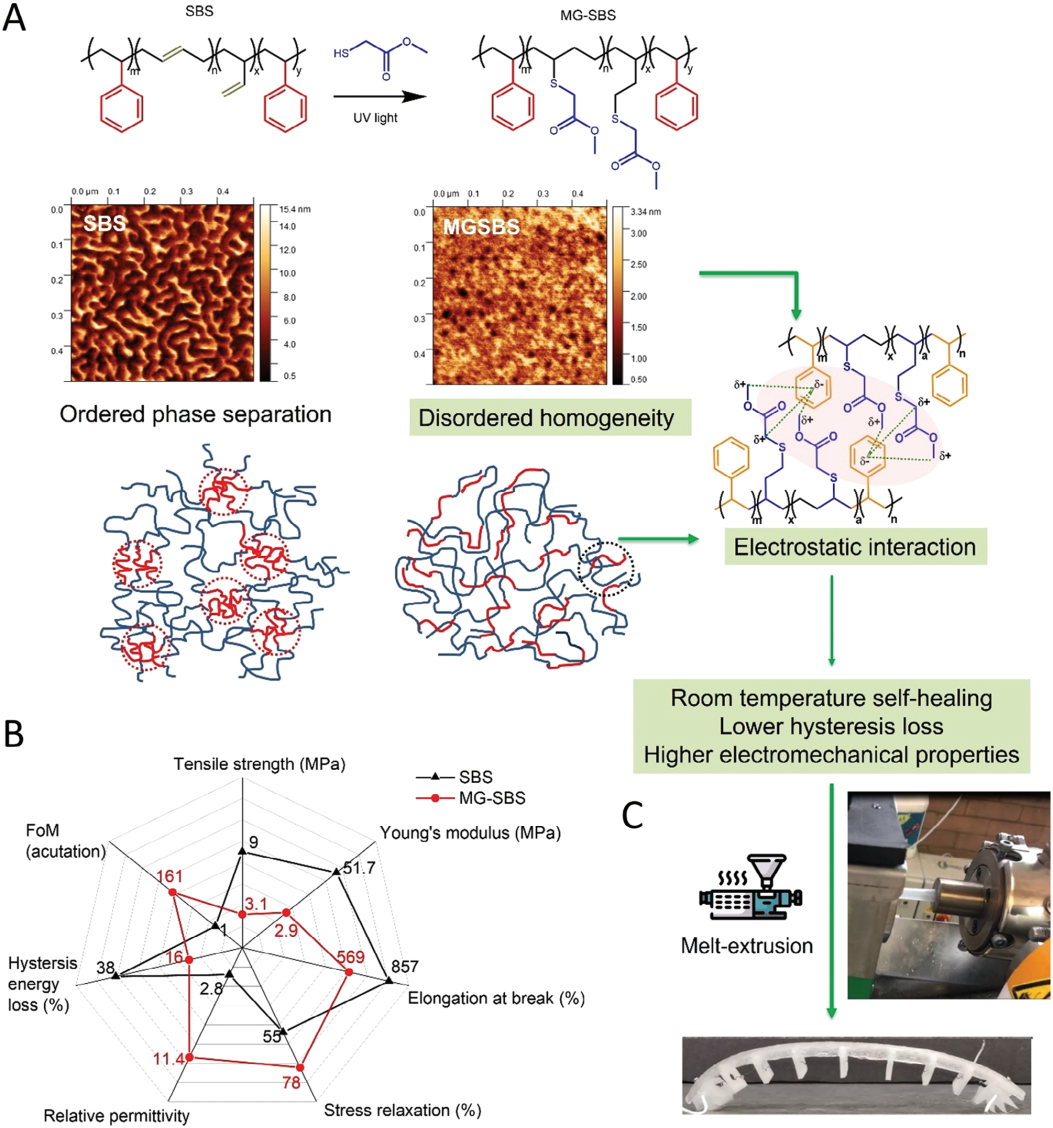
A) Modification of commodity SBS via one‐step thiol‐ene click chemistry to obtain MG‐SBS, AFM images showing the microstructure development,^[^ ^26^ ^]^ schematic showing the self‐healing mechanism of MG‐SBS; B) physical properties of SBS and MG‐SBS, and C) continuous melt‐extrusion of MG‐SBS to produce tubes for soft‐robot application.

**Figure** **1**A shows that the introduction of polar methyl thioglycolate to the butadiene blocks of SBS with a high grafting ratio of 98.5% induced a change in the phase morphology from order to disorder transition (AFM images^[^ ^26^ ^]^). This indicated that the polar groups have altered the intramolecular interactions and interrupted the clustering of the polystyrene hard domains. As characterized by Raman, real‐time FTIR, and SAXS in our previous work,^[^ ^24^ ^]^ the δ^+^ CH_2_ or δ^+^ CH_3_ group on either side of the ester of methyl thioglycolate is able to accept electron charge from the δ‐ aromatic center of styrene, i.e., CH···π interaction, which homogenizes the phase morphology from a sea‐island structure to a more disordered microstructure through the abundant electrostatic interactions along the polymer chains. As the electron density within the aromatic ring decreases, the HC‐CH aromatic bonds experience a weaker pull from the center of the ring, thereby increasing the bond length slightly. A similar interaction is observed in nature to give proteins their secondary structure.^[^ ^39^ ^]^ The self‐healing behavior of MG‐SBS originates from both the microstructural homogeneity and the dynamic electrostatic interactions among the polymer chains and is enhanced with the grafting ratio.^[^ ^24^ ^]^ Figure 1B provides a snapshot of the mechanical and electromechanical properties of SBS and MG‐SBS. The grafting of methyl thioglycolate increased the relative permittivity (*ε_r_*) of SBS from *ε_r_* = 2.8 to *ε_r_* = 11.4 at 10^3^ Hz in MG‐SBS. In addition, the MG‐SBS exhibited reduced stiffness and strength as compared to SBS, where the lower Young's modulus of 2.87 ± 0.6 MPa, tensile strength of 3.13 ± 0.12 MPa, and a strain at break of 569 ± 25.9% meet the mechanical property requirements of the high strain crawling robot, in particular, the reduced Young's modulus and lower hysteresis loss of MG‐SBS are advantageous for actuation. The stress relaxation of MG‐SBS is decreased to 78% of its maximum stress value when subjected to a fixed 100% elongation, which is higher than SBS of 55%, indicating the stronger interchain electrostatic interaction preventing polymer chain slippage, which does not exist in SBS.

Due to the thermoplastic nature of MG‐SBS, it can be continuously melt‐extruded into elastomer tubing at 170–180 °C (Figure 1C), where the tubes formed are able to preserve the ambient autonomous self‐healing properties. Due to the advantages of intrinsic healing, which can heal multiple times and does not require the use of encapsulants, and autonomous healing, which requires the application of additional external stimuli, we selected methyl thioglycolate. The grafting of methyl thioglycolate increased the relative permittivity (*ε*_r_) of SBS from *ε*_r_ = 2.8 to *ε*_r_ = 11.4 at 10^3^ Hz in MG‐SBS, while maintaining a low tan δ of 0.01 (where tan δ = dielectric loss/relative permittivity) at 10^3^ Hz. Meanwhile, MGSBS exhibited reduced stiffness and strength as compared to SBS, where the lower Young's modulus of 2.87 ± 0.6 MPa, tensile strength of 3.13 ± 0.12 MPa and a strain at break of 569 ± 25.9% is particularly attractive to the requirement the high strain crawling robot, where the reduced Young's modulus (from 51.7 ± 6.4 MPa for SBS to 2.87 ± 0.6 MPa for MG‐SBS) is advantageous for actuation.

Self‐healing of this material is likely to originate from either the δ+ CH_2_ or δ+ CH_3_ group on either side of the MG ester, which accepts electron density from the δ‐ aromatic ring, as shown in Figure 1. As the electron density within the aromatic ring decreases, the HC‐CH aromatic bonds experience a weaker pull from the center of the ring, increasing the bond length slightly. A similar interaction is observed in nature to give proteins their secondary structure.^[^ ^39^ ^]^ The mode of operation of the crawling robot leads to deformation of the main elastomer body to strain levels of ≈10%. Therefore, the key healing requirements are recovery of the stiffness of the material and the ability to recover the failure strain to a sufficient level.

### 2.2 Soft Self‐Healable Robot

**Figure 2:**
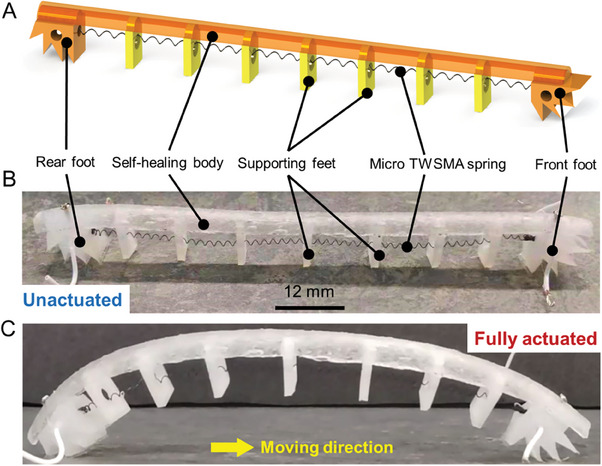
Architecture of the soft self‐healable robot. A) 3D model B) robot prototype C) fully actuated and deformed robot.

Inspired by the crawling mechanism of the caterpillar, which is also termed a two‐anchor crawling mechanism, the design and architecture of the self‐healable robot are shown in **Figure** **2**, which consists of a self‐healing body, a micro two‐way SMA (TWSMA) spring actuator, silicone‐based front and rear feet, and seven supporting feet. The self‐healing robot is designed to manage damage to its outer body not to its actuation system, since the outer body is most susceptible to damage from its local environment. The front and rear feet were cast using silicone rubber, and the supporting feet were 3D‐printed using polylactide (PLA). All feet were adhered to the self‐healing MG‐SBS body with the same gap distance of 15 mm. The length of the self‐healing body of the robot is 138 mm, with a “skin” thickness of 1 mm. A micro SMA spring actuator with a spring diameter of 1.5 mm and a length of 110 mm was arranged along with the body. The designed T‐shaped holes with a diameter of 3 mm in the front and rear feet are used to fix the TWSMA spring and the cable connected to the TWSMA spring, whereas a hole with a diameter of 4 mm on the supporting feet is present to arrange the TWSMA spring. When the TWSMA spring contracts, the distance between the front and rear feet decreases, and since the length of the self‐healing body is constant, a bending deformation is realized.

**Figure 3:**
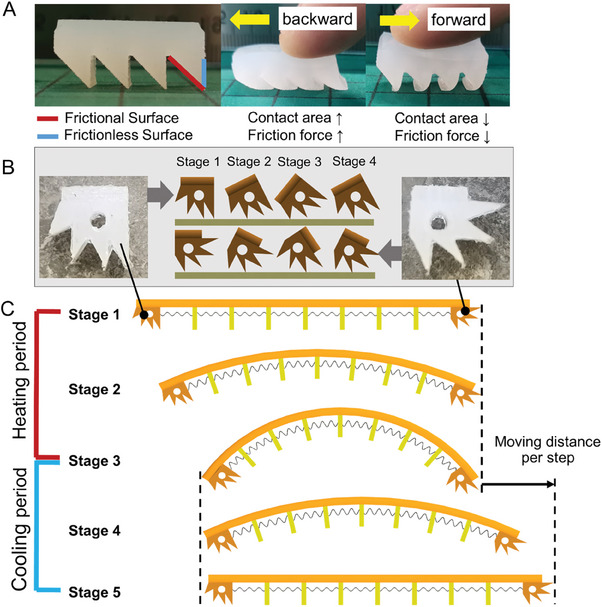
Mechanism of moving feet of the robot and robot locomotion A) Structure of single‐direction movement block and its mechanism when it moves backward and forward. B) Structures of the novel advanced rear and front feet and their statuses from the moving stage 1 to 4 during locomotion C) actuation mechanism of the self‐healable robot in a single locomotion step.

The self‐healable robot has two moving feet, the front and rear feet, and seven supporting feet. The moving feet enable the movement of the robot by spontaneously changing the friction force between the feet and the ground surface. The single‐direction mechanism of foot movement is shown in **Figure** **3**, where Figure 3A shows a wedge‐shaped structure with a frictionless surface and a frictional surface. The frictional surface is made using a silicone rubber with a high friction coefficient, while the frictionless surface with a low friction coefficient is realized by adhering transparent tape onto the silicone rubber surface. When the wedge block moves backward, the wedges deform, and the frictional surface makes contact with the ground, resulting in a significant increase in the contact area. As a result, a large friction force is generated and represents a high friction state. When the wedge block moves forward, only the small tip areas of the wedges deform. The contact surface with the ground is the frictionless surface and the contact area is relatively small, therefore, the generated friction force is small and represents a low friction state, as shown in Figure 3A. This use of single‐direction movement structure provides three advantages: i) the switching between the friction state can be realized by the internal actuation force, including the elastic potential energy of the TWSMA spring, the strain energy of the self‐healing body, and the gravitational potential energy of the robot, rather than the introduction of any external actuation forces; ii) the force required for switching the friction state is relatively small, and the forces produced by the robot itself are sufficiently large to change the friction state, including the elastic forces of both the SMA spring and the self‐healable robot body, and the gravity of the robot; iii) the switching time required to change the friction state is short, which can be neglected when controlling the robot. In summary, the friction force for a robot moving backward is much larger than that for moving forward, and the change of the friction force is only dependent on the moving direction. Therefore, by using a single‐directional movement mechanism, the self‐healable robot can effectively move forward.

To enable efficient movement, the advanced design of the robot feet is implemented on the self‐healing robot with an effective 0 to 90° range frictional contact surface, as shown in Figure 3B, while the simplified foot block is shown in Figure 3A can achieve single‐direction movement at a horizontal state. When the robot is actuated, the advanced design robot feet rotate from the horizontal state to another angle which, along with the bending of the body, efficiently generates frictional forces during movement to continuously drive the robot forward. Insufficient use of supporting feet can lead to collapse of the robot body due to gravity and the low stiffness of the self‐healing body. The use of too many supporting feet can lead to potential problems, including i) limited deformation due to the close arrangement of the feet and their self‐interference; and ii) the additional weight of the self‐healable robot that can limit robot movement. In our design, seven supporting feet were used based on the simulated results, which suggest the need for optimization between the movement flexibility and the robot weight. In addition, the TWSMA hole on the supporting feet should not interfere with the contraction and relaxation of the TWSMA spring, so the thickness of the supporting feet was designed as 2 mm, and the diameter of the hole is 4 mm, which is much larger than the diameter of the TWSMA spring (1.5 mm) to allow the free of movement.

Inspired by the crawling mechanism of the caterpillar, which is also termed a two‐anchor crawling mechanism, the actuation mechanism of the self‐healable robot in a single locomotion step is illustrated in Figure 3C. A single locomotion step can be divided into two periods, namely the TWSMA heating period and TWSMA cooling period. During the heating period, the TWSMA spring contracts and generates a force to pull the front and rear feet closer, so that the rear foot moves forward and the front foot keeps still due to the high friction force generated by the frictional surface of the wedge‐shape foot; see Stage 1 to Stage 3 in Figure 3C. The self‐healing tube is deformed into an inverted U shape, so the elastic potential energy of the TWSMA spring transforms into the gravitational potential energy of the robot and the strain energy of the self‐healing tube. During the cooling period, the TWSMA spring extends, and the self‐healing tube returns to its original shape as a result of the release of the gravitational potential energy and the strain energy; see Stage 3 to Stage 5 in Figure 3C. A force generated by these two energies pushes the front foot moving forward, while the rear foot keeps still due to the strong friction resistance. After a heating period and a cooling period of the TWSMA spring, the self‐healable robot can move forward over a distance, which is defined as the moving distance per step.

### 2.3 Robot Performance

**Figure 4:**
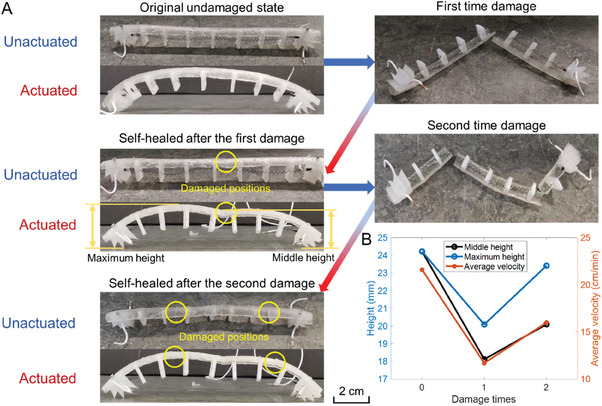
A) Schematic of the original and damaged robot and its self‐healing performance. B) Middle height, maximum height, and average velocity of the robot under different states.

To investigate the self‐healing capability of the robot, we cut and damaged the robot twice during characterization. The robot was autonomically healed successfully after the introduction of two damage events, as shown in **Figure** **4**A where the status and performance of the original and healed robot after the first damage and the second damage are shown. In the undamaged state, the average velocity of the original robot was 21.6 cm/min (= 18 mm ^−1^step) and the maximum height of the robot, as defined in Figure 4B, was 24.22 mm. The middle height equals the maximum height for an undamaged robot. The locomotion of the original robot is shown in Movie S1 (Supporting Information). The first damage was introduced by cutting with scissors in the middle position of the robot body. After the introduction of damage, the two parts of the robot were placed together for 24 h at room temperature to automatically self‐heal. The locomotion of the healed robot after the first damage is shown in Movie S2 (Supporting Information), where the average velocity decreases to 11.7 cm/min (= 9.75 mm ^−1^step). After healing from the initial introduction of damage, the self‐healable body forms an M shape when it is actuated by the micro SMA, instead of the original inverted U shape. This is because the elastic modulus of the material in the cutting location after healing is smaller than that of the original so the deformation at the healed location is larger than that of the rest of the self‐healable body. The middle and maximum heights are used to characterize the robot's performance, as shown in Figure 4B. The middle and maximum heights of the healed robot are 18.12 and 20.09 mm, respectively. The M shape deformation of the healed body decreases the maximum height and the average velocity of the robot, but the robot continues to function with good performance with good physical crawling heights of 75%–83% of the original height, and with an average crawling speed of 54% of the original speed.

To investigate the robustness and repeatability of the self‐healing performance, a second level of damage was conducted by introducing two scissor cuts at the positions of 1/4 and 3/4 of the self‐healable body. Three separated body parts were placed together for 24 h at room temperature for self‐healing. The locomotion of the robot after the second damage and 24 h self‐healing process are shown in Movie S3 (Supporting Information). The robot successfully recovered from the damage, where the average velocity was 16 cm/min (= 13.33 mm ^−1^step). The middle height of the robot is 20.1 mm, and the maximum height is 23.42 mm, which are very close to the original undamaged states. The robot functions with good physical crawling heights of 83%–96% of the original height, and with an average crawling speed of 74% of the original speed. When actuated, the robot is almost fully recovered to the original inverted U shape, as shown in Figure 4A.

### 2.4 Mechanical Properties of Micro SMA Spring Actuators

**Figure 5:**
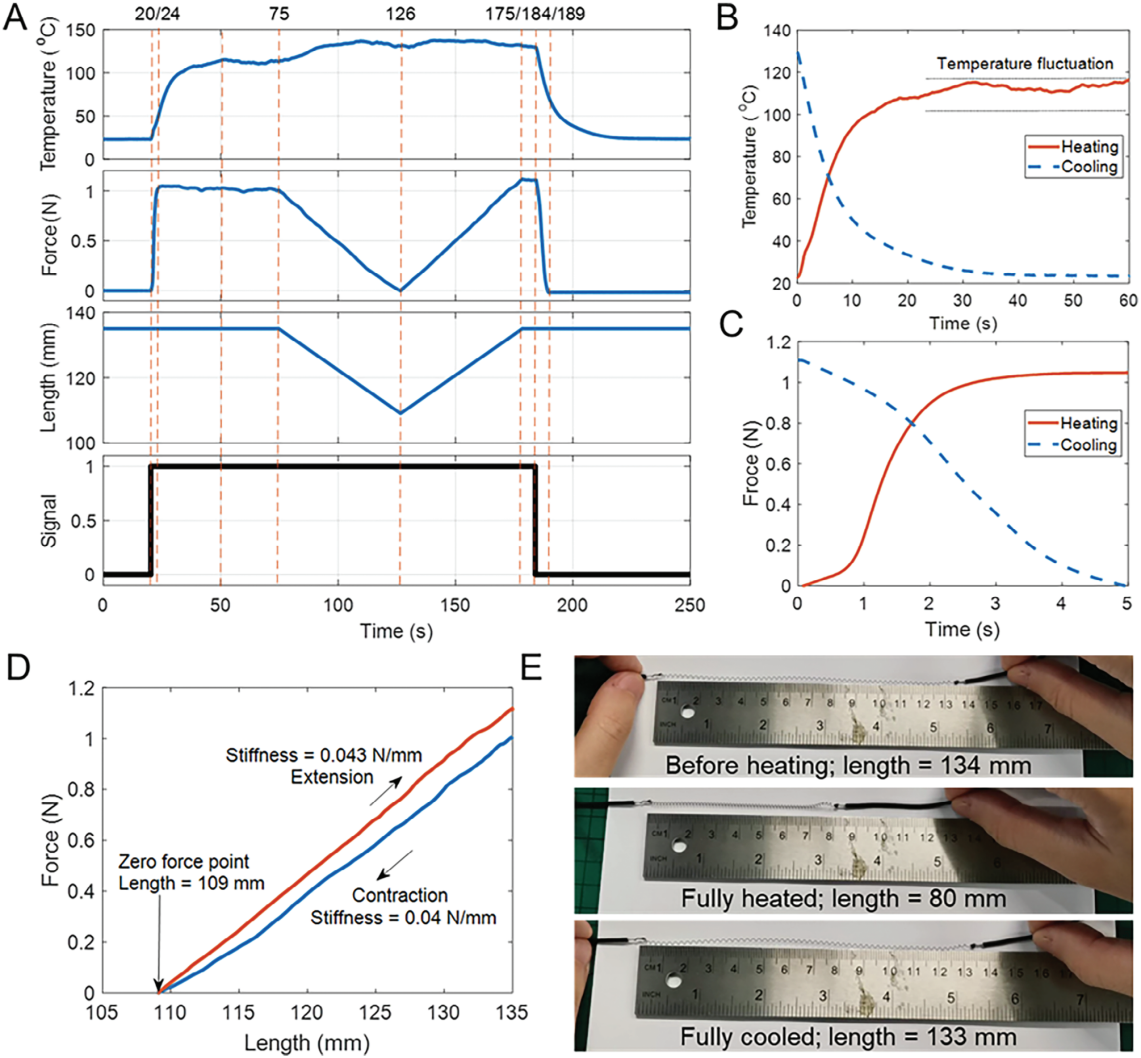
Properties of the micro SMA spring actuator. A) Temperature and force responses of the micro SMA spring actuator. Heating process (signal = 1) was achieved using a 5 V power supply and the cooling process (signal = 0) was achieved by placing the micro SMA spring at room temperature (≈23 °C). B) Temperature and force C) during the heating and cooling processes. D) Relationship between the force and length of the SMA spring during the compression and extension processes. E) Two‐way SMA effect and training preparation.

The mechanical properties of micro SMA spring actuators are shown in **Figure** **5**A, where the temperature, force, and length of the actuator were used to characterize the properties. The SMA length reflects the compression and extension processes of the actuator, whereas the signal indicates the heating (signal = 1) and cooling (signal = 0) states. The micro SMA spring actuator begins to heat at 20 s, and the temperature and force of the SMA spring are increased. The SMA spring needed to be heated for 30 s to reach the maximum temperature of 115 °C. The output force of the SMA spring increased from 0 to 1.04 N within 4 s and reached its steady state with a measured temperature of 55 °C. The SMA actuator was continually heated and began to compress at 75 s until reaching a zero‐output force at 126 s, and then returned to its original length at 175 s. After the compression and extension process, the micro SMA spring actuator stopped heating and started cooling at room temperature of 23 °C. During the cooling process, the output force decreased from 1.11 N to 0 in 5 s and the temperature dropped from 129 °C to 75 °C. The temperature of the SMA spring requires 60 s decreasing to return to room temperature.

The temperature and force during the heating and cooling processes are shown in Figure 5B,C. Compared to the cooling process, the temperature fluctuated to a greater extent during the heating process from 30 to 60 s, which was a result of the stress‐induced martensite (SIM) transformation.^[^ ^40^ ^]^ When the micro SMA is fully heated, its crystal structure transforms from martensite into austenite, and the transformation from austenite into martensite is achieved by cooling the SMA. The stress‐induced martensite transformation is an alternative method to achieve the structural transformation from austenite into martensite. During the heating process, the length of the micro SMA spring actuator was maintained constant in a tensile testing machine, which led to an increased output force, so that the actuator was stressed. While some martensite formation was induced by the stress‐induced transformation, the continual heating of the micro SMA actuator led to the martensite formed by the applied stress ultimately transforming to the austenite phase due to a temperature‐induced transformation. The competition between the stress‐induced transformation (from austenite to martensite) and the temperature‐induced transformation (from martensite to austenite) led to a fluctuation of temperature during the heating process, as shown in Figure 5B. Compared with the slow change of the temperature, the force changed rapidly during the heating and cooling process (see Figure 5C). The output force of the micro SMA spring actuators is related to the phase ratio between martensite and austenite phases of the SMAs, where the relationship between the spring length and force during compression and extension is shown in Figure 5D.

The stiffness of the SMA spring is 0.040 N mm^−1^ during compression and 0.043 N mm^−1^ during extension. The small difference in stiffness (0.003 N mm^−1^) is a result of the phase ratio of the martensite and austenite, which is dependent on the stress and temperature.^[^ ^41^ ^]^ The stress history experienced by the micro SMA spring actuator induces a stress‐induced martensite transformation, leading to the phase ratio difference and a very small difference in stiffness. The stress history is defined as the process of the stress suffered by the SMA spring from the initial start to the present. When the SMA spring is fully heated, the martensite is almost transformed into austenite, and the effect of temperature is reduced. However, the stiffness difference (0.003 N mm^−1^) is small and does not significantly affect the performance of SMA springs.

For the robot to operate repeatedly the shape memory effect needs to operate repeatedly and ideally provide a reversible spontaneous shape change during cooling and heating without any need to apply external stress. Figure 5E shows the two‐way properties of the micro SMA spring actuator, where the contraction and extension processes are shown in Movie S4 (Supporting Information). There was no external force acting on the micro SMA spring actuator during the processes, and the spring was free to move and unstressed. The phase transformation between the martensite and austenite phase were therefore a result of the change in temperature. The spring length at zero force is the length of the micro SMA spring without any external force acting on it, which is temperature‐dependent. Before the heating process, the spring length is 134 mm and during the heating process, the spring length contracts rapidly from 134 mm to 80 mm in 2 s due to the phase transformation from the martensite to austenite phase. During the cooling process, the spring length spontaneously extends to 133 mm in 10 s as it transforms from the austenite to martensite phase. The original length of the micro SMA spring actuator was almost recovered after being fully heated and cooled down to room temperature. During the two‐way SMA (TWSMA) training preparation, the micro SMA spring actuator generated permanent deformation by a plastic strain, which makes it possible to obtain a reversible spontaneous shape change during cooling and heating processes without any external stress. As a result, the TWSMA is able to memorize its shape at both high and low temperatures to provide a mechanism for robot motion. In Figure 5D, the contraction and extension of the micro SMA spring actuator were controlled by the tensile machine, resulting in external stress acting on it. After being fully heated, the spring length at zero force with an external stress is 109 mm, which is larger than 80 mm (see Figure 5E) without an external load. This length difference is caused by the stress history suffered by the micro SMA spring actuator. With regard to actuator performance, the effective working range of the actuator with stress was 109–135 mm, which is smaller than the stress‐free micro SMA (80–134 mm). A challenge of using a TWSMA is that the strain can rapidly deteriorate at high temperatures or when subjected to a high external force.^[^ ^42^ ^]^ Therefore, the micro SMA spring actuator was selected for use at low temperatures and low external force conditions within the soft robot.

### 2.5 Self‐Healable Robot Characterizisatation

#### 2.5.1 Robot Geometry Definition

**Figure 6:**
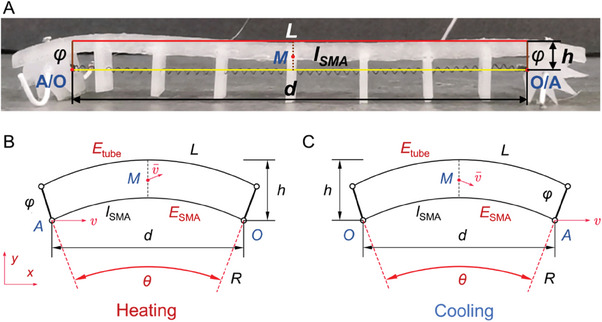
A) Geometry model of the self‐healable robot. Model definition of the situations when B) heating and C) cooling the TWSMA spring.

The analytical model of the self‐healable robot is shown in **Figure** **6**A. The length of the TWSMA spring is defined as *l_SMA_*, which is changed upon heating or cooling of the TWSMA spring. The body length of the self‐healable robot is constant and defined as *L*. The difference in height between the TWSMA spring and the self‐healing tube is constant and defined as *φ*. According to the actuation mechanism of the self‐healable robot in a single step, the front foot remains still on heating the TWSMA spring, whereas the rear foot remains still on cooling the TWSMA spring. Therefore, the origin at point *O* is defined as the bottom‐right or bottom‐left points when heating or cooling the TWSMA spring, respectively. A Cartesian coordinate system is established based on the origin, and another bottom point is defined as point *A*, as shown in Figure 6B,C. The horizontal distance between point *O* and point *A* is defined as *d*, whereas the height difference between point *O* and the vertex of the self‐healing tube is defined as *h*. The center of mass of the robot is set to the center of the rectangle as point *M*.

When the robot is actuated during the heating process, point A moves toward the *x*+ direction with a velocity of *v* and the rectangle body geometry deforms into an inverted “U” shape. To simplify the geometry, the deformed sides of both the TWSMA spring and the self‐healing body are assumed to be two concentric arcs. The radius and the angle of the TWSMA spring arc are *R* and *θ*. Therefore, we have

(1) $$R\left(\theta \right)=\frac{L}{\theta }-\varphi$$

(2) $$d\left(\theta \right)=2\left(\frac{L}{\theta }-\varphi \right)\sin\left(\frac{\theta }{2}\right)$$

(3) $$h\left(\theta \right)=\frac{L}{\theta }\left(1-\cos\frac{\theta }{2}\right)+\varphi \cos\frac{\theta }{2}$$

where *θ* ranges from 0 to π.

#### 2.5.2 Robot Dynamic Model

The model is analyzed using the law of conservation of energy. Four energies related to the robot locomotion are: i) the elastic potential energy of the TWSMA spring *E*_SMA_ ii) the strain energy of the self‐healing body *E*_tube_ iii) the gravitational potential energy *E*_g_, and iv) the kinetic energy *E*_v_. During the heating process, a part of the elastic potential energy of the TWSMA is transformed into strain energy and gravitational potential energy, and the remaining energy is converted into kinetic energy

(4) $$E_{v}=E_{SMA}-\left(E_{tube}+E_{g}\right)$$

Similarly, the relationship during the cooling process of the TWSMA spring is given as:

(5) $$E_{v}=E_{tube}+E_{g}-E_{SMA}$$

The kinetic energy *E*_v_ is related to the average velocity of the robot, as shown in Equation (6)

(6) $$E_{v}=\frac{1}{2}m{\bar{v}}^{2}$$

where *m* is the mass of the robot. The average velocity of the robot is:

(7) $$\bar{v}=\sqrt{\frac{2\left|E_{SMA}-\left(E_{tube}+E_{g}\right)\right|}{m}}$$

With the velocity and energy relationship derivations presented in the Supplementary Materials, the average velocity of the robot is given by:

(8) $$\begin{array}{c} \bar{v}=\theta^{\prime }\cdot \sqrt{\;\frac{{d^{\prime }}^{2}\left(\theta \right)}{4}+\frac{{h^{\prime }}^{2}\left(\theta \right)}{d^{2}{\left(h-\varphi \right)}^{2}}\;{\left[\;R^{2}\mathrm{arc}\sin\;\left(\;\frac{d}{2R}\;\right)-\frac{d}{2}\sqrt{R^{2}-\frac{d^{2}}{4}}\right]}^{2}} \end{array}$$

We define a function *f_v_* (θ)

(9) $$\begin{array}{c} f_{v}\left(\theta \right)=\sqrt{\;\frac{{d^{\prime }}^{2}\left(\theta \right)}{4}+\frac{{h^{\prime }}^{2}\left(\theta \right)}{d^{2}{\left(h-\varphi \right)}^{2}}\;{\left[\;R^{2}\mathrm{arc}\sin\;\left(\;\frac{d}{2R}\;\right)-\frac{d}{2}\sqrt{R^{2}-\frac{d^{2}}{4}}\right]}^{2}} \end{array}$$

As the angle *θ* increases during heating process but decreases during the cooling process, a sign function is used to calculate the direction of *θ*. It is defined the angle *θ* equals 0 when the robot is unactuated. Combining Equations (7–, 9), an ordinary differential equation (ODE) for the dynamic model of the self‐healable robot can be established

(10) $$\begin{array}{c} \theta^{\prime }=\frac{\bar{v}}{f_{v}\left(\theta \right)}=\frac{sgn\left(E_{SMA}-\left(E_{tube}+E_{g}\right)\right)}{f_{v}\left(\theta \right)}\sqrt{\;\frac{2\left|E_{SMA}-\left(E_{tube}+E_{g}\right)\right|}{m}} \end{array}$$

### 2.6 Robot Elastic Potential Energy

The elastic potential energy of the robot TWSMA spring body is

(11) $$E_{SMA}=\frac{1}{2}k\Delta x^{2}=\frac{1}{2}k{\left(l_{SMA}-l_{0}\right)}^{2}$$

where *k* is the stiffness and *l*_0_ is the length of spring at zero force. According to Equation (11), the elastic potential energy is related to the real‐time length of the TWSMA spring *l*_SMA_ (*l_SMA_* = θ*R*), and the material properties of the TWSMA spring. The properties of the TWSMA spring depend on the ratio between the martensite and austenite phases, which is a function of temperature and stress.^[^ ^43^, ^44^ ^]^ Here we considered that the properties of the TWSMA spring are dominated by the temperature with a neglected effect on stress. For a TWSMA spring with a constant *l*_SMA_, increasing *k* and decreasing *l*_0_ results in a higher force. During the heating process, the TWSMA spring temperature increases from room temperature (≈23 °C) to the maximum (102 °C) and the output force increases from 0 to the maximum (1.04 N); the condition of cooling is the opposite (see Figure 5). Therefore, we have

(12) $$\lim_{T\to T_{\max}}F_{SMA}=F_{\infty },\lim_{T\to T_{room}}F_{SMA}=0$$

The related stiffness *k* and the zero‐force length *l*_0_ at the maximum force are *k*_∞_ and *l*_0,∞_. The output force of the TWSMA spring at the temperature *T* is *F*_T_, the related stiffness is *k*_T_ and the zero‐force length is *l*_0,T_. A linear relationship is assumed and given by Equation (13):

(13) $$\frac{F_{T}}{F_{\infty }}=\frac{k_{T}}{k_{\infty }}=\frac{L-l_{0,T}}{L-l_{0,\infty }}$$

During the heating process, the heating temperature is controlled to allow only a part of the martensite phase to transform into austenite. The experimental force and temperature results are in Figure 5B,C were used to establish the relationship between the force and temperature. They are separately fitted by two following models,

(14) $$F\left(t\right)=A\left[1-\frac{1}{1+{\left(t/t_{0}\right)}^{p}}\right]$$

(15) $$T\left(t\right)=T_{0}+T_{1}\left(1-e^{-t/t_{1}}\right)$$

The fitting parameters of force are *A* = 1.052 N, *t*_0_ = 1.3 s, and *p* = 4.158, whereas that of temperature are *T*_0_ = 23 °C, *T*_1_ = 96.63 °C, and *t*_1_ = 8.304 s. Before conducting the cooling process, both martensite and austenite phases exist in the TWSMA spring body, and the austenite phase transforms into martensite upon cooling. However, the transformation kinetics of the phase ratio is difficult to accurately predict during real‐time motion. After the TWSMA spring was fully heated, only the austenite existed and the output force of the TWSMA spring during the cooling process decreased from the maximum of 1.11 N to zero in 5 s (see Figure 5C). The temperature of the TWSMA at 5 s is 72 °C, which is sufficient to transform martensite into austenite during heating (see Figure 5B). Here, it is assumed that the change rate of the spring force during cooling *k*_cooling_ is defined as:

(16) $$\frac{dF}{dt}=k_{cooling}$$

where *k*_cooling_ is assumed to be a constant and fitted by the force result during the cooling process, which is −0.2217 N/s.

### 2.7 Strain Energy

When the self‐healing body deforms, it stores strain energy that is related to the deformation of the robot body. Due to the large deformation and the nonlinearity of the constitutive model of the self‐healing tube, it is difficult to solve the analytical solutions for the strain energy. Finite element analysis models were developed for analyzing the strain energy of the self‐healing body. A two parameters Mooney‐Rivlin model was used as the constitutive model of the self‐healing tube, and the model is given as,

(17) $$W=C_{10}\left(I_{1}-3\right)+C_{01}\left(I_{2}-3\right)$$

**Figure 7:**
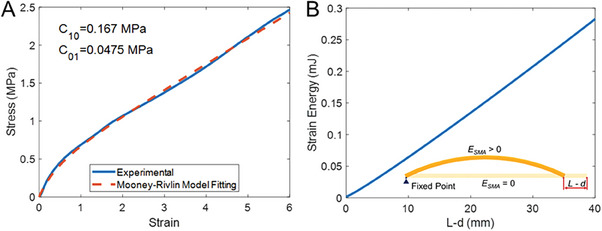
A) Experimental results and fitting curves based on the two‐parameter Mooney‐Rivlin model. B) Finite element analysis model and strain energy of the self‐healing robotic body.

The parameters *C*_10_ and *C*_01_ were fitted by uniaxial extension experiment data. The equation of uniaxial extensional stress is

(18) $$\sigma_{11}=2\left(C_{10}+\frac{C_{01}}{\lambda }\right)\left(\lambda -\frac{1}{\lambda^{2}}\right)$$

where *λ* is the ratio of the final and initial lengths in the principal directions. The fitting results of the parameters *C*_10_ and *C*_01_ are 0.167 and 0.0475 MPa, as shown in **Figure** **7**A. The finite element model (FEM) was conducted in ANSYS 2021 R1, where the *fixed point* and the *free‐moving point* of the robot body are defined as shown in Figure 7B. FEM modeling is

(19) $$E_{\mathrm{tube}}=f_{tube}\left(d\right)$$

The strain energy used in the dynamic model is calculated by using spline interpolation based on the FEM results.

### 2.8 Gravitational Potential Energy

The gravitational potential energy is calculated by using the average rising height of the robot

(20) $$E_{g}=mg\bar{h}$$

The average raising height during the heating or cooling process is,

(21) $$\bar{h}=\frac{1}{d}\int_{-d}^{0}y_{p}dx_{p}=\frac{R^{2}}{d}\mathrm{arc}\sin\left(\frac{d}{2R}\right)-\frac{\sqrt{4R^{2}-d^{2}}}{4}$$

### 2.9 Model Evaluation

**Figure 8:**
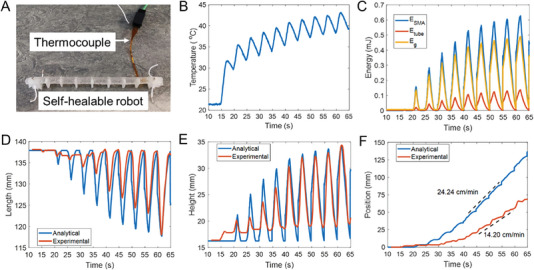
Analytical and experimental robot energies and mechanical properties A) Thermocouple attached to the self‐healable robot and B) the temperature of the TWSMA spring. C) Elastic potential energy ESMA, strain energy Etube, and gravitational potential energy *E*_g_ calculated by the dynamic model. D) Height, E) length, and F) position validated in experiments.

The model of the self‐healable robot is evaluated analytically and experimentally. The length of the self‐healing robotic body *L* is 110 mm, the high difference *φ* is 7 mm, and the mass of the robot is 4 g, as shown in **Figure** **8**A. For the TWSMA spring properties, the stiffness *k*_∞_ is 0.04 N mm^−1^ and the zero‐force length *l*_0,∞_ is 89 mm after the TWSMA is fully heated. A pulse width modulation (PWM) waveform with a period of 5 s consisting of a heating time of 1.8 s and a cooling time of 3.2 s is used to control the TWSMA spring. A K‐type polyimide flat film thermocouple was attached to the TWSMA spring to measure the temperature, as shown in Figure 8A and in Movie S5 (Supporting Information). Figure 8B shows the experimental temperature which was used for the ordinary differential equation (ODE) dynamic model to calculate the elastic potential energy. The TWSMA spring was arranged at room temperature before 15 s. After 15 s, the TWSMA spring was actuated by using the pulse‐width modulation (PWM) driven signal, and the temperature started to increase. The temperature output performs a zigzag shape with a period of 5 s. The elastic potential energy ESMA, strain energy Etube, and gravitational potential energy *E*_g_ obtained by calculating ODE dynamic model are shown in Figure 8C. The kinetic energy *E*_v_ of the robot is small and was not plotted. For the self‐healable robot, the elastic potential energy *E*_SMA_ is the dominant energy. During the heating process, most of the elastic potential energy *E*_SMA_ is transformed into the gravitational potential energy *E*_g_, the minority of energy is transformed into the strain energy *E*_tube_, and limited energy is transformed into the kinetic energy *E*_v_.

The simulated and experimental results of robot performance including robot height, length, and position during the movement are shown in Figure 8D–F. The TWSMA spring was actuated at 15 s, and the maximum temperature in the first PWM‐driven period (15–20 s) was 32 °C, which is insufficient to transform the martensite phase into the austenite phase, resulting in the force generated by the TWSMA spring is insufficient to actuate the soft‐healable robot. After the second PWM period (20–25 s), the change in the energies and robot motion can be observed. All three energies *E*_g_, *E*_SMA,_ and Etube increased with the temperature, and the difference between elastic potential energy *E*_SMA_ and gravitational potential energy *E*_g_ became larger; see Figure 8C. This is because of the decrease in robot length, and more elastic potential energy ESMA is transformed into strain energy *E*_tube_. As shown in Figure 8D,E, the simulated robot height and length agreed well with the experimental results, which can effectively predict the motion trend and characteristics. Some differences occurred at the minimum and maximum heights and lengths, and this is because the deformation of silicone moving feet was not considered and included in the robot dynamic model. The robot's position is defined based on the center point of the robot body. As shown in Figure 8F, the robot was accelerated from 0–40 s, and the moving velocity tended to remain steady after 40 s. From 40 s – 60 s, the simulated average moving velocity is 24.24 cm/min, while the experimental average moving velocity is 14.2 cm/min. The velocity difference is caused by using the thin and light flat film thermocouple which can hinder the movement of the robot during the experiments. For comparison, the average velocity of the robot achieved in the robot's original state without attaching the film thermocouple to the robot is 21.6 cm/min, which is similar to the simulated results. The small velocity difference could be a result of a skidding effect. During the heating or cooling processes, the status of the front or rear feet is considered stationary; however the friction force of the silicone rubber is not infinite, so the front and rear feet may skid, depending on the surface, when the robot moves. The success of the model provides analytical tools to analyze the motion of the robot theoretically and accurately without the need to conduct more complex case‐dependent computational finite element models.

### 2.10 Self‐Healing Efficiency

The mechanical and electrical properties of the MG‐SBS self‐healing tube and its repeated recoveries were examined. Self‐healing efficiency can be quantified by comparing the healed properties with the original properties. We examine here the self‐healing efficiency of the materials after both mechanical damages. A tensile bar specimen was cut in half, and then we placed the two surfaces back in contact and left at room temperature for 72 h. The recovered mechanical properties are elongation at break 116%, stress 0.8 MPa, and stiffness of 10.85 MPa, as compared to the original specimen which has an elongation at break of 569%, a stress of 3.13 MPa and a stiffness of 2.87 MPa, a self‐healing efficiency of 39% was obtained. The self‐healing capacity ensures that our new self‐healable robot is able to recover from mechanical damage with good performance, in particular the stiffness and elongation to break.

**Figure 9:**
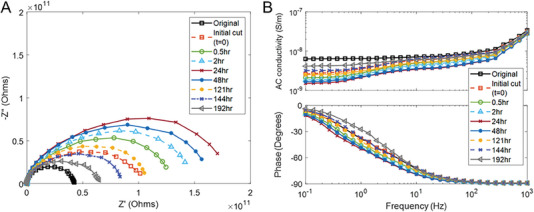
A) Complex impedance response of MG‐SBS with healing time B) Frequency dependence of AC conductivity (upper image) and phase angle (lower image) with healing time.

**Figure** **9**A shows the complex impedance response of the initial (uncut) material and its response with healing time after the cut surfaces were placed in contact with each other. The elastomer exhibits a classical semi‐circle impedance response which represents a parallel arrangement of a resistor (*R*) and capacitance (*C*), where the intersection of the semi‐circle arc with the Z’‐axis at a low frequency corresponds to the overall resistance. Figure 9B also shows the AC conductivity and phase angle with frequency for the uncut elastomer, and during healing. At low frequencies (0.1–10 Hz) the AC conductivity is relatively frequency independent, indicating the material is behaving as a conductor; this agrees with the phase angle approaching 0° in this low‐frequency range. At higher frequencies (>10 Hz), the material behaves as a capacitor, where the AC conductivity becomes frequency dependent and the phase angle approaches −90°, since an AC current lags AC voltage by 90°. As there is a limited difference between the capacitive impedance response of the uncut material and healed material at high frequencies (Figure 9B), it can be assumed that the capacitance, and therefore permittivity, does not change significantly during healing and any changes in impedance are dominated by changes in conductivity and electrical transport across the healing interface.

Figure 9A indicates that the uncut material has the smallest semi‐circle intersection point and resistance, which corresponds to the highest conductivity in Figure 9B. On placing the cut surfaces in contact, there is a rapid recovery of the low frequency (0.1 Hz) conductivity from 6.3 × 10^−5^ S m^−1^ to 2.6 × 10^−5^ m^−1^, and this corresponds to an increase in the intersection of the semi‐circle to the Z’‐axis in Figure 9A (“initial cut”). During the initial 24 h of self‐healing, the intersection points of the semi‐circle increase, where there is a small decrease in the low‐frequency conductivity to 1.5 ×10^−5^ m^−1^. The initial rapid recovery is likely to be due to rapid electrostatic interactions during the healing processes of surface rearrangement and surface approach, with subsequent wetting of the surfaces by a phase of lower conductivity greater than the initial material as the healing process continues. As the healing time progresses from 24 h to 192 h (8 days) the semi‐circle intersection point then decreases (Figure 9A) and the AC conductivity approaches that of the initial uncut material to a value of 4.5 × 10^−5^ m^−1^. This may be due to longer healing time allowing greater diffusion and randomization of material at the fracture site, so that the healed regions become more representative of the initial material, as the mechanism of Wool. The initial rapid recovery of electrical response, in particular the AC conductivity and phase angle, indicates that the material provides sufficient self‐healing via electrostatic bonding and a degree of diffusion to achieve the necessary degree of self‐healing for recovery of mechanical properties.

## 3 Discussion

We have created a soft crawling robot that can self‐heal autonomously to its full performance at room temperature after being significantly damaged. The innovative design of the robot integrates a micro TWSMA spring actuation system and a self‐healing dielectric elastomer MG‐SBS.

The thermoplastic nature of the MG‐SBS elastomer material allows continuous manufacturing of intrinsically self‐healing MG‐SBS tubes by scalable industrial melt extrusion process, which expands the applicability of self‐healing robots for practical applications. The introduction of organic polar groups to commodity SBS thermoplastic elastomers increased the relative permittivity (*ε*_r_) of SBS from *ε*_r_ = 2.8 to *ε*_r_ = 11.4 at 10^3^ Hz, meanwhile, the highly grafted polar groups along the polymer backbones enhanced the intramolecular interactions and interrupted the polystyrene clustering, so that induced the phase morphology transition from original ordered structure to more disorder morphology. This explains the enhanced extensibility (569 ± 25.9%), reduced Young's modulus (2.87 ± 0.6 MPa), and autonomous ambient self‐healing via the abundant electrostatic interactions among the polymer chains. The intrinsic self‐healing nature of the material as a result of electrostatic interactions allows repeated recovery of the required elastic modulus and strain to failure at room temperature after 24 h, with a low hysteresis loss. The rapid self‐healing is also observed in terms of recovery of electrical properties, in particular the rapid recovery of AC conductivity and phase.

A two‐anchor crawling mechanism is applied to the robot for locomotion. Taking advantage of the controllable heating and cooling processes of TWSMA spring actuation system, the movement and speed of the robot are controllable by adjusting the PWM control signal. In addition, the advanced optimized moving feet were developed to maximize the moving efficiency. The structure and the geometry of the robot feet can be applied to a variety of soft crawlers in different scales.

The performance of the self‐healable robot was validated in experiments. The measured deformation (maximum and middle heights) and crawling speed of the robot resulting from heating and cooling period of TWSMA agree with the analytical modeling results. Some expected velocity discrepancies can be caused by the attachment of the thermocouple on the robot during the experiments, and the skidding effect of the feet on the experiment platform surface. The self‐healing capacity of the robot was verified by healing from significant repetitive cutting damages. The robot was cut into half and three separate parts and fully recovered after 24 h at room temperature. Small weak scars can be seen after cutting and healing due to the change of modulus at the cutting positions but they do not significantly affect the robot performance. The self‐healing tube and the robot are easy to conduct and cost‐efficient to provide autonomous healing without the need for external stimuli. This material combination and integration enable high‐performance soft robots with autonomic self‐healing capability without external stimulus, which is novel in the field. We created the analytical models for the self‐healable robot which can effectively predict the characteristics and dynamics of the robot, without conducting complex computation‐inefficient finite element models. This can be a valuable tool for the future design, analysis, and optimization of soft crawlers.

In summary, this study presents a new methodology for the design and actuation of self‐healable robots. The MGSBS‐based geometry can be used for a variety of soft robotic applications and increases the life span of the soft components and robots, while the TWSMA spring actuator can be applied as a new integrated actuation system to soft robots using its two‐way memory effect. Our demonstration has successfully proven the concepts that can be developed for high technology readiness levels applications, such as exploration robots and industrial robots under uncertain and extreme environments, which can improve the sustainability of robots and generate research impact.

## 4 Experimental Section

### Micro SMA Spring Actuator

**Figure 10:**
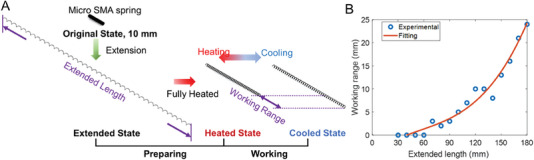
A) Preparation of a two‐way micro SMA actuator and B) The relationship between extended length and working range.

The Micro SMA could be categorized into two characteristics: one‐way SMA (OWSMA) and two‐way SMA (TWSMA). The OWSMA retained a deformation after removing the external force and recovered to its initial state when being heated. The TWSMA could memorize its state at both low and high temperatures. Considering that the force generated by the gravitational potential energy and the strain energy of the self‐healable robot was insufficient to extend and deform the OWSMA spring during the cooling period (see stage 3 to stage 5 in Figure 3C), the TWSMA spring was determined to use to create the robot to mimic the locomotion of caterpillars. This was a first in the design of soft self‐healing robots. Perkins concluded that the TWSME was a result of a macroscopic non‐uniform residual stress field, so a non‐uniform plastic deformation was a necessary condition to obtain a TWSME.^[^ ^45^ ^]^ Schroeder proposed that the growth of detwinned martensite was related to characteristics of the two‐way shape memory.^[^ ^46^ ^]^ Wada and Liu proved that the dislocation structures created by martensite pre‐deformation were beneficial for generating a better TWSME experimentally.^[^ ^47^ ^]^ Therefore, a large pre‐deformation method was used to prepare the TWSMA spring in other work (see **Figure** **10**A). The original length of the SMA spring was 10 mm. The original SMA spring was pre‐extended at room temperature, and the length of the extended spring was defined as extended length. After pre‐extension, the extended SMA spring was heated without an external load for 1 min by applying a 5 V power supply; during this time, the SMA spring contracted significantly. Finally, the SMA spring was cooled to room temperature, and the TWSMA spring was realized. The length difference between heated and cooled states was defined as the working range. The relationship between the extended length and the working range is shown in Figure 10B. With an increase in the extended length, the working range increases significantly. The data was fitted by using a power model

(22) $$y=ax^{b}$$

where *a* and *b* are 2.5 × 10^−5^ and 2.65. The extended length of the TWSMA spring was 180 mm.

### Self‐Healing Materials—Materials

Styrene‐butadiene‐styrene (SBS), Vector 8508A, was a linear triblock copolymer from Dexco. It had a 29 wt.% styrenic content, a diblock content of <1 wt.%, a density of 0.41 g/cm^3^ (ASTM D1895), a melt flow rate of 12 g 10 min^−1^ (ASTM D1238) and recommended processing conditions of between 180 °C – 225 °C. General reagents methyl thioglycolate (MG, 95%) was purchased from Sigma‐Aldrich.

### Self‐Healing Materials—Manufacturing Self‐Healing Tube

The methyl thioglycolate‐modified SBS (MG‐SBS) was detailed in our previous work.^[^ ^24^ ^]^ The MGSBS pellets were fed into a single‐screw extrusion machine (Collin Germany) tubes for melt‐extrusion of elastomer tubes. The processing temperature was set as 150, 170, 180, and 175 °C from the feeding zone to the die (inner diameter of 6 mm, external diameter of 7 mm), extrusion rate was 30 rpm.

### Experimental Preparation and Measurement—Robot Feet Preparation and Fabrication

The single‐direction robotic feet were manufactured by molding. The molds and cores were 3D‐printed using PLA, and the detailed design can be found in Supplementation. After assembling the mold, the mixing silicone liquid rubber (Ecoflex 00–30, Smooth‐on) was poured into the mold and placed at room temperature at 23 °C. The silicone rubber was cured completely after 4 h. Finally, the single‐direction feet were removed from the mold.

### Experimental Preparation and Measurement—Tensile Testing and Temperature Measurement of Micro SMA

Tensile testing was conducted using a tensile testing machine Instron 3369. During the tensile test experiments, four steps were performed: i) The SMA spring was heated by a 5 V power supply and kept at the same length for 1 min. ii) The SMA spring was compressed with a speed of 0.5 mm ^−1^s until its output force reached zero. iii) The SMA spring was extended to its original length with a speed of 0.5 mm ^−1^s. iv) The SMA spring was kept at the same length and cooled for 2 min.

A K‐type polyimide flat film thermocouple (TC Direct) was attached to the TWSMA spring to measure its real‐time temperature. A thermocouple module (NI‐9213, National Instruments) carried by a compact‐DAQ chassis (USB‐9162, National Instruments) was used to record the temperature data. The data acquisition program was based on LabVIEW, and the sampling frequency was 20 Hz.

### Experimental Preparation and Measurement—SMA Robot Controller

An Arduino microcontroller board (Mega‐2560) was used to generate the PWM signal with a period of 5 s. The output signal drives a relay module to switch the heating and cooling modes for the TWSMA spring.

### Experimental Preparation and Measurement—Electrical Characterisation

The electrical properties of the materials during self‐healing were determined using a Solartron 1260 Impedance Analyser with a Solartron 1296 Dielectric Interface. The sample was electroded with silver paint and the complex impedance of the materials was measured from 0.1 Hz to 103 Hz at 3 V peak to peak in an air‐conditioned laboratory at 16 °C. The sample was initially tested, then cut using a clean razor blade, and tested immediately after placing the two cut surfaces back in contact with each other. The sample was then periodically tested as it self‐healed for up to 192 h.

The AC conductivity (admittance) was determined using Equation (23),

(23) $$\sigma =\frac{Z^{\prime }}{{Z^{\prime }}^{2}+{Z^{\prime \prime }}^{2}}\cdot \frac{t}{A}$$

where *Z*′ and *Z*″ are the real and imaginary parts of the impedance, respectively, *A* is the area of the materials and *t* is the thickness. The phase angle (*θ*) between current and voltage was determined from Equation (24):

(24) $$\theta =\tan^{-1}\left(Z^{\prime \prime }/Z^{\prime }\right)$$

## Conflict of Interest

The authors declare no conflict of interest.

## Author Contributions

M.P. and C.W. conceptualized the idea. X.R.L., M.P., and C.W. designed the methodology. The investigation involved X.R.L., C.G.Y, X.L.G., and C.B. M.P. managed funding acquisition and project administration. M.P., C.W., and C.B. did the supervision. X.R.L., M.P., C.W., and C.B. wrote the first draft, and X.R.L., C.G.Y., M.P., C.W., and CB reviewed and edited it.

## Supporting information

Supporting Information

Supplemental Video 1

Supplemental Video 2

Supplemental Video 3

Supplemental Video 4

Supplemental Video 5

## Data Availability

The data that support the findings of this study are available from the corresponding author upon reasonable request.

## References

[1] D. Rus , M. T. Tolley , Nature 2015, 521, 467. 26017446 10.1038/nature14543

[2] C. Laschi , B. Mazzolai , M. Cianchetti , Sci. Rob. 2016, 1, eaah3690. 10.1126/scirobotics.aah3690 33157856

[3] C. Lee , M. Kim , Y. J. Kim , N. Hong , S. Ryu , H. J. Kim , S. Kim , Int. J. Control Autom. 2017, 15, 3.

[4] M. Calisti , G. Picardi , C. Laschi , J. R. Soc. Interface 2017, 14, 20170101. 28539483 10.1098/rsif.2017.0101 PMC5454300

[5] M. Sitti , Nat. Rev. Mater. 2018, 3, 74.

[6] R. F. Shepherd , F. Ilievski , W. Choi , S. A. Morin , A. A. Stokes , A. D. Mazzeo , X. Chen , M. Wang , G. M. Whitesides , Multigait soft robot, PNAS, Washington, DC, 2011, 108, 20400. 10.1073/pnas.1116564108 PMC3251082 22123978

[7] M. T. Tolley , R. F. Shepherd , B. Mosadegh , K. C. Galloway , M. Wehner , M. Karpelson , R. J. Wood , G. M. Whitesides , Soft Robot 2014, 1, 213.

[8] N. W. Bartlett , M. T. Tolley , J. T. B. Overvelde , J. C. Weaver , B. Mosadegh , K. Bertoldi , G. M. Whitesides , R. J. Wood , Science 2015, 349, 161. 26160940 10.1126/science.aab0129

[9] E. W. Hawkes , L. H. Blumenschein , J. D. Greer , A. M. Okamura , Sci. Robot. 2017, 2, eaan3028. 33157883 10.1126/scirobotics.aan3028

[10] S. I. Rich , R. J. Wood , C. Majidi , Nat. Electron. 2018, 1, 102.

[11] S. Terryn , J. Brancart , D. Lefeber , G. Van Assche , B. Vanderborght , Sci. Robot. 2017, 2, eaan4268. 33157852 10.1126/scirobotics.aan4268

[12] G. Li , X. Chen , F. Zhou , Y. Liang , Y. Xiao , X. Cao , Z. Zhang , M. Zhang , B. Wu , S. Yin , Y. Xu , H. Fan , Z. Chen , W. Song , W. Yang , B. Pan , J. Hou , W. Zou , S. He , X. Yang , G. Mao , Z. Jia , H. Zhou , T. Li , S. Qu , Z. Xu , Z. Huang , Y. Luo , T. Xie , J. Gu , et al., Nature 2021, 591, 66. 33658693 10.1038/s41586-020-03153-z

[13] R. P. Wool , Soft Matter 2008, 4, 400. 32907199 10.1039/b711716g

[14] M. D. Hager , P. Greil , C. Leyens , S. Van Der Zwaag , U. S. Schubert , Adv. Mater. 2010, 22, 5424. 20839257 10.1002/adma.201003036

[15] Y. Yang , M. W. Urban , Chem. Soc. Rev. 2013, 42, 7446. 23864042 10.1039/c3cs60109a

[16] T.‐P. Huynh , P. Sonar , H. Haick , Adv. Mater. 2017, 29, 1604973. 10.1002/adma.201604973 28229499

[17] R. A. Bilodeau , R. K. Kramer , Front. Robot. AI 2017, 4.

[18] Y. Zhang , H. Khanbareh , J. Roscow , M. Pan , C. Bowen , C. Wan , Matter 2020, 3, 989.

[19] S. Terryn , J. Langenbach , E. Roels , J. Brancart , C. Bakkali‐Hassani , Q.‐A. Poutrel , A. Georgopoulou , T. G. Thuruthel , A. Safaei , P. Ferrentino , T. Sebastian , S. Norvez , F. Iida , A. W. Bosman , F. Tournilhac , F. Clemens , G. Van Assche , B. Vanderborght , Mater. Today 2021, 47, 187.

[20] Y.u J. Tan , G. J. Susanto , H. P. Anwar Ali , B. C. K. Tee , Adv. Mater. 2021, 33, 2002800. 10.1002/adma.202002800 33346389

[21] Y. Gai , H.u Li , Z. Li , Small 2021, 17, 2101383. 10.1002/smll.202101383 34288411

[22] J. Ekeocha , C. Ellingford , M. Pan , A. M. Wemyss , C. Bowen , C. Wan , Adv. Mater. 2021, 33, 2008052. 10.1002/adma.202008052 34165832

[23] E. Roels , S. Terryn , F. Iida , A. W. Bosman , S. Norvez , F. Clemens , G. Van Assche , B. Vanderborght , J. Brancart , Adv. Mater. 2022, 34, 2104798. 10.1002/adma.202104798 34610181

[24] C. Ellingford , R. Zhang , A. M. Wemyss , C. Bowen , T. Mcnally , L. Figiel , C. Wan , Acs. Appl. Mater. Inter. 2018, 10, 38438. 10.1021/acsami.8b13785 30360080

[25] Y. Zhang , C. Ellingford , R. Zhang , J. Roscow , M. Hopkins , P. Keogh , T. Mcnally , C. Bowen , C. Wan , Adv. Funct. Mater. 2019, 29, 1808431.

[26] C. Ellingford , R. Zhang , A. M. Wemyss , Y. Zhang , O. B. Brown , H. Zhou , P. Keogh , C. Bowen , C. Wan , Acs. Appl. Mater. Inter. 2020, 12, 7595. 10.1021/acsami.9b21957 31944651

[27] R. F. Shepherd , A. A. Stokes , R. M. D. Nunes , G. M. Whitesides , Adv. Mater. 2013, 25, 6709. 24123311 10.1002/adma.201303175

[28] S. Terryn , J. Brancart , E. Roels , G. Van Assche , B. Vanderborght , IEEE Robot. Autom. Mag. 2020, 27, 44. 10.1089/soro.2019.0081 32160110

[29] E. Roels , S. Terryn , J. Brancart , R. Verhelle , G. Van Assche , B. Vanderborght , Soft Robot 2020, 7, 711. 32160110 10.1089/soro.2019.0081

[30] T. J. Wallin , J. H. Pikul , S. Bodkhe , B. N. Peele , B. C. Mac Murray , D. Therriault , B. W. Mcenerney , R. P. Dillon , E. P. Giannelis , R. F. Shepherd , J. Mater. Chem. B 2017, 5, 6249. 32264440 10.1039/c7tb01605k

[31] K. Yu , A.n Xin , H. Du , Y. Li , Q. Wang , NPG Asia Mater 2019, 11, 7.

[32] Y. Zhang , X.‐Y.u Yin , M. Zheng , C. Moorlag , J. Yang , Z. L. Wang , J. Mater. Chem. A 2019, 7, 6972.

[33] B. Zhang , W. Zhang , Z. Zhang , Y.‐F. Zhang , H. Hingorani , Z. Liu , J. Liu , Q.i Ge , Acs. Appl. Mater. Inter. 2019, 11, 10328. 10.1021/acsami.9b00359 30785262

[34] E. F. Gomez , S. V. Wanasinghe , A. E. Flynn , O. J. Dodo , J. L. Sparks , L. A. Baldwin , C. E. Tabor , M. F. Durstock , D. Konkolewicz , C. J. Thrasher , Acs. Appl. Mater. Inter. 2021, 13, 28870. 10.1021/acsami.1c06419 34124888

[35] E. Acome , S. K. Mitchell , T. G. Morrissey , M. B. Emmett , C. Benjamin , M. King , M. Radakovitz , C. Keplinger , Science 2018, 359, 61. 29302008 10.1126/science.aao6139

[36] Y.e Tian , J. Liu , W. Wu , X. Liang , M. Pan , C. Bowen , Y. Jiang , J. Sun , T. Mcnally , D. Wu , Y. Huang , C. Wan , Adv. Intell. Syst. 2022, 4, 2100239.

[37] W. Tang , C. Zhang , Y. Zhong , P. Zhu , Y.u Hu , Z. Jiao , X. Wei , G. Lu , J. Wang , Y. Liang , Y. Lin , W. Wang , H. Yang , J. Zou , Nat. Commun. 2021, 12, 2247. 33854071 10.1038/s41467-021-22391-x PMC8046788

[38] J.‐N. Ma , Y.‐L. Zhang , Y.‐Q. Liu , D.‐D. Han , J.‐W. Mao , J.‐R. Zhang , W.‐C. Zhao , H.‐B. Sun , Sci. Bull. 2022, 67, 501. 10.1016/j.scib.2021.11.015 36546171

[39] C. H. Suresh , N. Mohan , K. P. Vijayalakshmi , R. George , J. M. Mathew , J. Comput. Chem. 2009, 30, 1392. 19037862 10.1002/jcc.21162

[40] D. Fahr , Metall. Trans. 1971, 2, 1883.

[41] R. Stalmans , J. Van Humbeeck , L. Delaey , Acta metallurgica et materialia 1992, 40, 2921.

[42] J. M. Jani , M. Leary , A. Subic , M. A. Gibson , Mater. Des. 2014, 56, 1078.

[43] H. Tobushi , K. Tanaka , JSME Int. J. I‐SOLID M. 1991, 34, 83.

[44] J. O. Alcaide , L. Pearson , M. E. Rentschler , in 2017 IEEE Int. Conf. Robot. Automat. (ICRA) , 2017, 4338.

[45] J. Perkins , Scripta Metall. Mater. 1974, 8, 1469.

[46] T. A. Schroeder , C. M. Wayman , Scripta Metall. Mater. 1977, 11, 225.

[47] K. Wada , Y. Liu , J. Alloys Compd. 2008, 449, 125.

