# Supplementary material for: Soft Self‐Healing Robot Driven by New Micro Two‐Way Shape Memory Alloy Spring
Source: Adv Sci (Weinh). 2023 Nov 20;11(2):2305163. doi: 10.1002/advs.202305163 (PMC10787064; doi:10.1002/advs.202305163)
Supplement: Supplementary file 1 — Supporting Information [file ADVS-11-2305163-s003.pdf]

ADVANCED SCIENCE Open Access

Supporting Information

for *Adv. Sci.*, DOI 10.1002/advs.202305163

Soft Self-Healing Robot Driven by New Micro Two-Way Shape Memory Alloy Spring

Xianrong Liang, Chenggang Yuan, Chaoying Wan, Xiaolong Gao, Chris Bowen and Min Pan**

Supporting Information

Soft self-healing robot driven by new micro two-way shape memory alloy spring

Xianrong Liang,[1] Chenggang Yuan,[1] Chaoying Wan[2], Xiaolong Gao[3], Chris Bowen[1], Min Pan[1]**

[1]Department of Mechanical Engineering, University of Bath; Bath, UK
E-mail: mp351@bath.ac.uk

[2]International Institute for Nanocomposites Manufacturing (IINM), WMG, University of Warwick, CV4 7AL, UK.
E-mail: Chaoying.Wan@warwick.ac.uk

[3]College of Mechanical and Electrical Engineering, Beijing University of Chemical Technology, Beijing 100029, China.

1. Robot dynamic model

As shown in Equation (7) of the main text, the average velocity is the robot is:

$$\bar{v} = \sqrt{\frac{2\left|E_{SMA} - \left(E_{tube} + E_g\right)\right|}{m}} \tag{S1}$$

Here, the arc OA (see **Figure.6**) is extracted to calculate the robot's velocity, as shown in **Figure. S1**. To establish the Cartesian coordinate, the origin is set on the point *O*, and the point *A* is located on the *x*-axis. Point *Q* is the highest point of the arc OA. According to the Cartesian coordinate, the average velocity is given as

$$\bar{v} = \sqrt{\bar{v}_x^2 + \bar{v}_y^2} \tag{S2}$$

The *x*- and *y*-directional average velocities are also related to the change rates of d and h, respectively, as shown below:

$$\begin{cases} v_d = \dfrac{\partial d}{\partial t} = \dfrac{\partial d}{\partial \theta} \cdot \dfrac{\partial \theta}{\partial t} = d'(\theta) \cdot \theta' \\ v_h = \dfrac{\partial h}{\partial t} = \dfrac{\partial h}{\partial \theta} \cdot \dfrac{\partial \theta}{\partial t} = h'(\theta) \cdot \theta' \end{cases} \tag{S3}$$

Where

$$d'(\theta)=\left(\frac{L}{\theta}-\varphi\right)\cos\frac{\theta}{2}-\frac{2L}{\theta^2}\sin\frac{\theta}{2} \tag{S4}$$

$$h'(\theta)=\frac{1}{2}\left(\frac{L}{\theta}-\varphi\right)\sin\frac{\theta}{2}-\frac{L}{\theta^2}\left(1-\cos\frac{\theta}{2}\right) \tag{S5}$$

As shown in **Figure. S1**, the maximum *x*-direction velocity $v_{x,max}$ on the arc OA is on the point A, whereas the maximum y-direction velocity $v_{y,max}$ on the arc OA is on the point *Q*. Point *P* is an arbitrary point on the arc OA. An assumption is made that the relation between the velocity components on the point *P* and the maximum velocity on the arc OA is given by

$$\frac{v_{px}}{v_{x,\max}}=\frac{x_p}{x_a},\frac{v_{py}}{v_{y,\max}}=\frac{y_p}{y_q} \tag{S6}$$

Where

$$\begin{cases} v_{x,\max}=v_d, v_{y,\max}=v_h \\ x_a=\mp d, y_q=h-\varphi \end{cases} \tag{S7}$$

The position of the arbitrary point *P* satisfies the following circle equation

$$\left(x_p\pm\frac{d}{2}\right)^2+\left(y_p+\sqrt{R^2-\frac{d^2}{4}}\right)^2=R^2 \tag{S8}$$

Thus, we have

$$y_p=\sqrt{R^2-\left(x_p\pm\frac{d}{2}\right)^2}-\sqrt{R^2-\frac{d^2}{4}} \tag{S9}$$

From Equation (S7) to Equation (S9), the ± sign is determined by the state of the heating or cooling of the TWSMA spring. According to Equation (S6), the *x* and *y* components of velocity at the arbitrary point *P* are:

$$v_{px}=\mp\frac{v_d}{d}\cdot x_p \tag{S10}$$

$$v_{py}=\frac{v_h}{h-\varphi}\cdot y_p=\frac{v_h}{h-\varphi}\left[\sqrt{R^2-\left(x_p\pm\frac{d}{2}\right)^2}-\sqrt{R^2-\frac{d^2}{4}}\right] \tag{S11}$$

Therefore, the *x*- and *y*-directional average velocities are:

$$\bar{v}_x=\frac{1}{d}\int_0^d v_{px}dx_p=\frac{v_d}{2} \tag{S12}$$

$$\bar{v}_y=\frac{1}{d}\int_0^d v_{py}dx_p=\pm\frac{v_h}{d(h-\varphi)}\left[R^2\arcsin\left(\frac{d}{2R}\right)-\frac{d}{2}\sqrt{R^2-\frac{d^2}{4}}\right] \tag{S13}$$

According to Equation (S2), the average velocity of the robot is:

$$\bar{v} = \theta' \cdot \sqrt{\frac{d'^2(\theta)}{4} + \frac{h'^2(\theta)}{d^2(h-\varphi)^2}\left[R^2 \arcsin\left(\frac{d}{2R}\right) - \frac{d}{2}\sqrt{R^2 - \frac{d^2}{4}}\right]^2} \tag{S14}$$

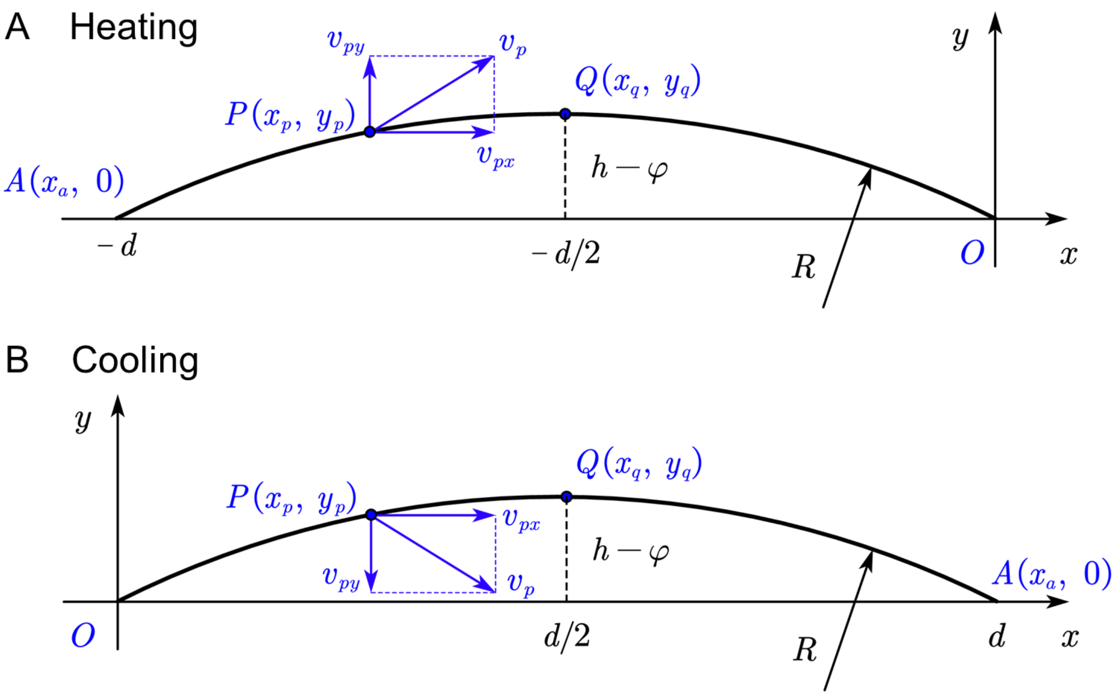

Figure. S1. Definition of point P and its related velocity during **(A)** heating and **(B)** cooling the TWSMA spring.

2. FEM modelling

The strain energy of the self-healing tube is calculated by Finite Element Method (FEM), which was conducted in ANSYS 2021 R1. The geometry of the self-healing tube was modeled as a semi-tube with a length of 120 mm, and inner and outer diameters of 5 mm and 7 mm, respectively (see **Figure. S2 (A)**). The tube material is considered to follow Mooney-Rivlin model and the model parameters can be found in **Figure. 7(A)**. The bottom flat surface is placed on the x-z surface. As shown in **Figure. S2(B)**, two fixed edges are at the left side of the tube (highlighted by green), while two free moving edges are at the right side of the tube (highlighted by yellow). The free moving edges move in the z+ direction, and the displacement of free moving edges is from 0 to 60 mm with a step of 0.01 mm. The simulated strain energy distribution at the final state is shown in **Figure. S2(C)**. The total strain energy at each displacement step was calculated, and the relation between displacement and strain energy is shown in **Figure. 7(B)**.

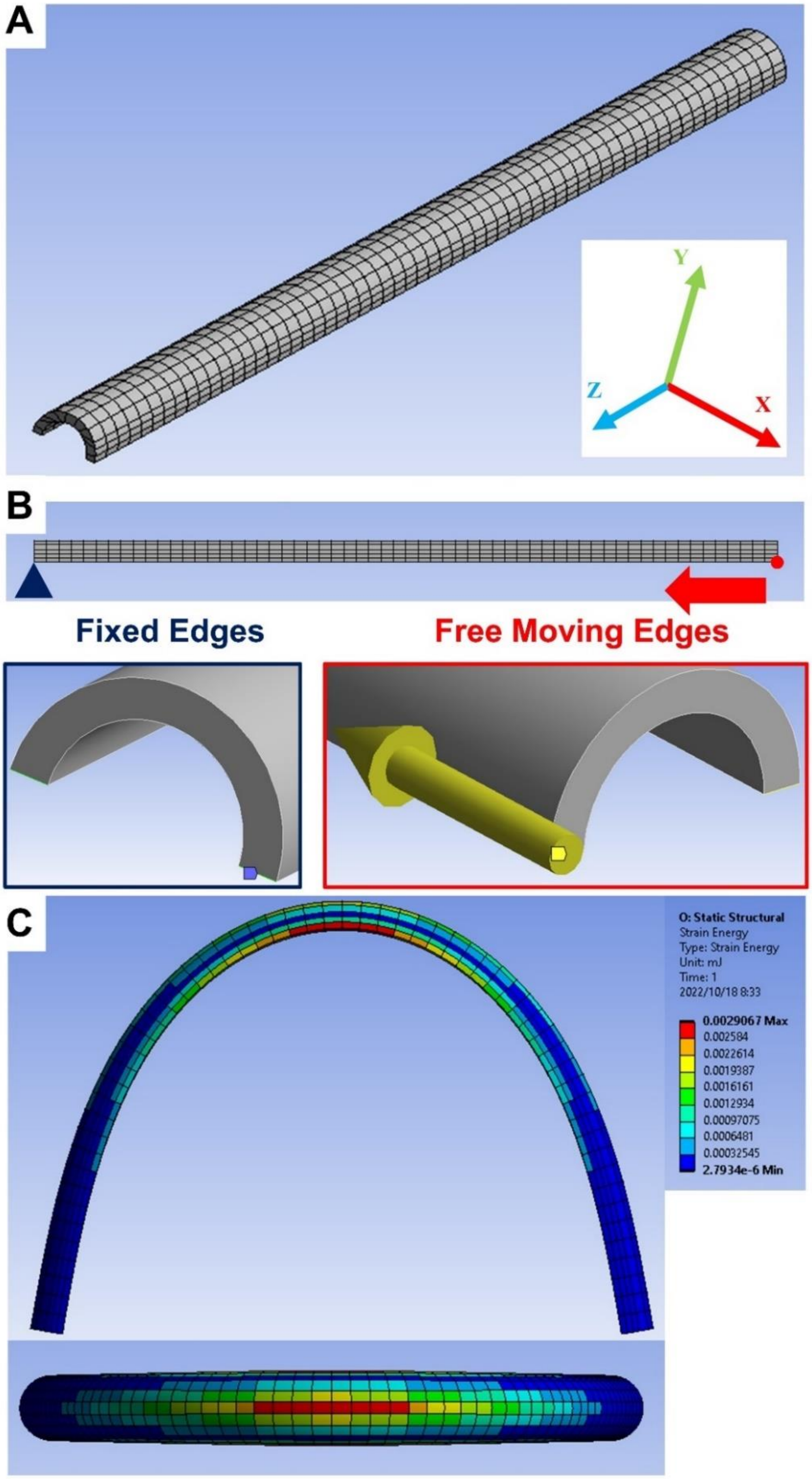

Figure. S2. (A) Geometry model, **(B)** boundary conditions and **(C)** simulated strain energy distribution of the self-healing tube.

Movies S1-S5
